# Targeting Gremlin 1 Prevents Intestinal Fibrosis Progression by Inhibiting the Fatty Acid Oxidation of Fibroblast Cells

**DOI:** 10.3389/fphar.2021.663774

**Published:** 2021-04-22

**Authors:** Yang Yang, Qi-Shan Zeng, Min Zou, Jian Zeng, Jiao Nie, DongFeng Chen, Hua-Tian Gan

**Affiliations:** ^1^ Department of Gastroenterology and the Center of Inflammatory Bowel Disease, West China Hospital, Sichuan University, Chengdu, China; ^2^ Lab of Inflammatory Bowel Disease, Clinical Institute of Inflammation and Immunology, Frontiers Science Center for Disease-related Molecular Network, West China Hospital, Sichuan University, Chengdu, China; ^3^ Department of Gastroenterology, Daping Hospital, Army Medical University, Chongqing, China; ^4^ Department of Gastroenterology, Chongqing Traditional Chinese Medicine Hospital, Chongqing, China; ^5^ Department of Geriatrics and National Clinical Research Center for Geriatric, West China Hospital, Sichuan University, Chengdu, China

**Keywords:** intestinal fibrosis, gremlin 1, fibroblast cell, fatty acid oxidation, VEGFR2

## Abstract

Intestinal fibrosis is a consequence of continuous inflammatory responses that negatively affect the quality of life of patients. By screening altered proteomic profiles of mouse fibrotic colon tissues, we identified that GREM1 was dramatically upregulated in comparison to that in normal tissues. Functional experiments revealed that GREM1 promoted the proliferation and activation of intestinal fibroblast cells by enhancing fatty acid oxidation. Blocking GREM1 prevented the progression of intestinal fibrosis *in vivo.* Mechanistic research revealed that GREM1 acted as a ligand for VEGFR2 and triggered downstream MAPK signaling. This facilitated the expression of FAO-related genes, consequently enhancing fatty acid oxidation. Taken together, our data indicated that targeting GREM1 could represent a promising therapeutic approach for the treatment of intestinal fibrosis.

## Introduction

Intestinal fibrosis, a common complication of inflammatory bowel disease (IBD) (Leake, 2015), is characterized by exaggerated scar tissue formation in the colon mucosa (Rieder and Fiocchi, 2009) resulting from intestinal damage induced by chronic inflammation (Lawrance et al., 2017). Despite great progress in the treatment of IBD, the incidence of intestinal fibrosis has not markedly reduced (Su et al., 2016; Vítek and Tiribelli, 2020), indicating that the development of new targeted therapies or effective interventions for intestinal fibrosis are urgently needed.

Intestinal fibroblasts, the major effector cells of gastrointestinal fibrosis (Weder et al., 2018), mainly account for the synthesis of several extracellular matrix proteins. This includes collagens, vimentin, and fibronectin (Hagenlocher et al., 2017). During chronic inflammation processes, local gut mesenchymal cells are activated and proliferated. This leads to enhanced deposition of extracellular-matrix proteins. Previous studies reported that resident fibroblasts can be activated by inflammation-related proteins (Lawrance et al., 2001), including the growth factors IGF-I, FGF, and PDGF, and the pro-inflammatory cytokines IL-1*β* and TNF-*α* (Simmons et al., 2002). This results in expansion of fibroblast cell numbers and fibroblast differentiation. In addition, colonic transforming growth factor (TGF)-*β*1 overexpression results in fibroblast accumulation and bowel wall thickening (Vallance et al., 2005). Apart from cytokines, growth factors, and chemokines, local fibroblasts resident in the colon can also respond to other biological mediators (Fichtner-Feigl et al., 2007; Burke et al., 2008). Thus, screening new biological mediators may provide potential therapeutic targets.

GREM1, encoded by the *GREM1* gene (Mezzano et al., 2018), is an important protein secreted in development, including organ formation, physique development, and tissue differentiation. GREM1 is considered to be an antagonist of the bone morphogenetic protein (BMP) family members and binds to BMP2 to regulate the BMP signaling pathway (Hsu et al., 1998). Later studies revealed that GREM1 can act as a vascular endothelial growth factor receptor 2 (VEGFR2) ligand in vascular endothelial cells to regulate angiogenesis (Mitola et al., 2010). Additionally, GREM1 has been reported to be both tumor promotive and suppressive in cancer. Elevated GREM1 expression is associated with favorable outcomes in several malignancies, including colon adenocarcinoma (Nyqvist et al., 2017) and gastric cancer (Honma et al., 2018). On the other hand, increased GREM1 expression facilitates the proliferation of cancer cells in lung adenocarcinoma and glioma. However, the role of GREM1 in intestinal fibrosis remains unknown.

In this study, we found that protein levels of GREM1 were dramatically increased in fibrotic colon. The loss and gain of function experiments revealed that GREM1 promoted intestinal fibroblast cell proliferation by enhancing fatty acid oxidation. Further mechanistic studies revealed that GREM1 could activate VEGFR2 and trigger downstream MAPK signaling. This facilitates FAO-related gene expression, consequently enhancing fatty acid oxidation and results in fibroblast cell proliferation and activation. Taken together, these results provide evidence that GREM1 could be a potential target for intestinal fibrosis therapy.

## Materials and Methods

### Animal Experiments

All animal experiments were approved by the Institutional Animal Care and Use Committee (IACUC) of the Army Medical University, and were authorized by the Institutional Animal Care and Use Committee of the Laboratory Animal Center of Daping Hospital, Army Medical University. Mouse intestinal fibrosis was induced by the administration of 2.5% dextran sulfate sodium (DSS) in tap water for 5 days followed by 1°week of normal drinking water for three cycles (Speca et al., 2016). The mice were sacrificed at nine weeks after DSS treatment. Intestinal tissues were formalin-fixed and embedded in paraffin for further analysis.

### Cell Lines

Human intestinal fibroblast cell lines CCD-18Co and CCD-112Co were maintained at the Daping Hospital, Army Medical University. Cells were cultured in Dulbecco’s modified Eagle’s medium (DMEM) supplemented with 10% fetal bovine serum (FBS, Gibco), 100°U/ml penicillin and 100 μg/ml streptomycin at 37°C in a 5% CO_2_ atmosphere. Exogenous GREM1, used for cell treatment, purchased from R&D system (cat#:5190-GR-050). STR identification was conducted to exclude exogenic cell contamination.

### Cell Proliferation Assays

Enumeration of fibroblasts was performed using Cell Counting Kit-8 (CCK8, NCM), according to the manufacturer’s instructions. Cells were cultured in 96-well plates at 3,000 cells/well. Serum conditions included no serum added to the culture medium, and other components were consistent with full medium. At the indicated time points, the cell culture medium was changed to a CCK8 buffer, and after 90 min incubation at 37°C, under 5% CO_2_, OD_450_ values were measured to determine cell viability.

### Transfection and RNA Interference

siRNA transfection was conducted using Lipofectamine® 2000 (Invitrogen) according to the manufacturer’s instructions. siRNA was purchased from GenePharma and Shanghai BioTend Biotech Co. Total cellular RNA was extracted after 48 h of transfection. SiRNA sequences are presented in Supplementary Material.

### RNA Isolation and Q-PCR

Total RNA was isolated using Trizol reagent (Takara, 9109) and reverse transcription was performed using PrimeScript kits (Takara, RR037A). To detect gene expression, RT-qPCR assays were performed using SYBR Premix Ex Taq (Roche) using the recommended thermal settings. Relative mRNA expression was analyzed using the 2 (-ΔΔCt) method and normalized to that of 18S ribosomal RNA. PCR primer sequences are presented in Supplementary materials.

### Immunoblotting

Whole cell lysates were prepared with RIPA lysis buffer (share-bio Tech) and membrane proteins were extracted using a Membrane and Cytosol Protein Extraction Kit (Beyotime). Quantification of protein concentrations was performed using a BCA protein assay kit (Thermo Fisher Scientific Proteins were resolved by 4–20% SDS-PAGE and transferred to NC membranes (Whatman), then antibody and ECL methods were employed to measure protein expression levels. Primary antibodies used were as follows: anti- GREM1 (R&D Systems, AF956, 1:1,000), anti-phospho-VEGF Receptor 2 (Tyr1175) (CST, #3770, 1:1,000), anti-VEGF receptor 2 (CST, #2479, 1:1,000), anti-phospho-MEK1/2 (Ser217/221) (CST, #3958, 1:1,000), anti-MEK1/2 (CST, #4694, 1:1,000), anti-phospho-p44/42 MAPK (Erk1/2) (Thr202/Tyr204) (CST, #9101,1:1,000), and anti-p44/42 MAPK (Erk1/2) (CST, #4695, 1:1,000). The secondary antibodies were anti-rabbit (share-bio, SB-AB0101, Abways Technology, AB0151), anti-mouse (share-bio, SB-AB0102, Yeasen Biotech Co., 33206ES60), and anti-goat (Immunoway Biotech Co., RS2123; Abcam, ab175776).

### Sample Preparation and Proteomic Analyses

The fibrosis colon and control group samples were grinded by liquid nitrogen into cell powder and then transferred to a 5-ml centrifuge tube. After that, four volumes of lysis buffer (8 M urea, 1% protease Inhibitor Cocktail) was added to the cell powder, followed by sonication three times on ice using a high intensity ultrasonic processor (Scientz). The remaining debris was removed by centrifugation at 12,000 g at 4°C for 10 min. As for proteomic analyses, trypsin was added into the protein solution at 1:50 trypsin-to-protein mass ratio for the first digestion overnight and 1:100 trypsin-to-protein mass ratio for a second 4 h-digestion. Then, the tryptic peptides were dissolved in 0.1% formic acid (solvent A), directly loaded onto a home-made reversed-phase analytical column (15-cm length, 75 μm i. d.). The gradient was comprised of an increase from 6 to 23% solvent B (0.1% formic acid in 98% acetonitrile) over 26 min, 23–35% in 8 min and climbing to 80% in 3 min then holding at 80% for the last 3 min, all at a constant flow rate of 400 nL/min on an EASY-nLC 1000 UPLC system.

The peptides were subjected to NSI source followed by tandem mass spectrometry (MS/MS) in Q ExactiveTM Plus (Thermo) coupled online to the UPLC. The electrospray voltage applied was 2.0 kV. The m/z scan range was 350–1800 for full scan, and intact peptides were detected in the Orbitrap at a resolution of 70,000. Peptides were then selected for MS/MS using NCE setting as 28 and the fragments were detected in the Orbitrap at a resolution of 17,500. A data-dependent procedure that alternated between one MS scan followed by 20 MS/MS scans with 15.0 s dynamic exclusion. Automatic gain control (AGC) was set at 5E4. Fixed first mass was set as 100 m/z. The resulting MS/MS data were processed using Maxquant search engine (v.1.5.2.8). Tandem mass spectra were searched against human uniprot database concatenated with reverse decoy database. Trypsin/*P* was specified as cleavage enzyme allowing up to four missing cleavages. The mass tolerance for precursor ions was set as 20 ppm in First search and 5 ppm in Main search, and the mass tolerance for fragment ions was set as 0.02 Da. Carbamidomethyl on Cys was specified as fixed modification and acetylation modification and oxidation on Met were specified as variable modifications. FDR was adjusted to <1% and minimum score for modified peptides was set >40.

### Histology Score

We scored all histological sections of the colonic samples in double-blind, according to the presence of ulcerations (0 = absent, 1 = small ulcers, 2 = big ulcers), degree of inflammation (0 = absent, 1 = mild, 2 = moderate, and 3 = severe), depth of the lesions (0 = absent, 1 = lesions extending in the submucosa, 2 = lesions in the muscularis propria, and 3 = lesions in the serosa), and degree of fibrosis (0 = absent; 1 = mild, 2 = moderate, and 3 = severe). The sum of these scores was expressed as total microscopic score.

### Immunofluorescence and Immunohistochemistry

Cells cultured on Millicell® EZ SLIDE (MILLIPORE) were fixed with 4% paraformaldehyde for 15 min and permeabilized with 0.1% Triton X-100 for 1 min at room temperature. After blocking with BSA (Sangon Biotech), samples were incubated with the primary antibody (1:200) overnight at 4°C, and the secondary antibody–labeled with fluorescein (1:200)–for 1 h at room temperature. Finally, DAPI was used to counterstain nuclei and the sample images were captured using a confocal microscope. For immunofluorescent staining of living cells to locate membrane proteins, cells were rinsed with cold PBS, followed by incubation with primary and secondary antibodies (1:500) for 1 h at 4°C. Next, the cells were fixed with 4% paraformaldehyde and stained with DAPI. For immunohistochemistry, slides were deparaffinized in xylene, and then antigen retrieval was performed using a citrate antigen retrieval solution. Endogenous peroxidase activity was blocked through the addition of hydrogen peroxide. After blocking with 10% BSA at room temperature, slides were incubated with primary antibodies at 4°C overnight. Following 1 h incubation with HPR-conjugated secondary antibody (anti-mouse, 1:500, Jackson ImmunoResearch, 115–035–003 or rabbit secondary antibodies 1:500 Jackson ImmunoResearch, 111–035–003), slides were developed in DAB (CST, 8059) and counterstained with hematoxylin.

### BODIPY Staining

BODIPY staining was conducted according to standard procedures. Briefly, control and GREM1 treated fibroblasts were incubated with the BODIPY dye solution (Servicebio, GP1104) at room temperature in the dark for 1 h, followed by nuclear staining with DAPI and examination by confocal microscopy. Cells in five images of each sample were counted using ImageJ software.

### Lipid Lysis Product Analysis

The amounts of acylcarnitine and acetyl-CoA were measured according to a previously reported method (Cheng et al., 2020). Briefly, serum-free cultured fibroblasts were treated with GREM1 and were collected and homogenized. They were then suspended in chloroform/methanol (1:1), and analyzed by mass spectrometry. The amounts of acylcarnitine and acetyl-CoA were normalized to the total protein content of fibroblast cells.

### Public Dataset Bioinformatic Analysis

SRA datasets (SRP077046 and SRP100787) containing data for tissues from both healthy controls and CD and UC patients. The mRNA expression level of *GREM1* was compared in both healthy controls and CD and UC patients.

### Seahorse Assays

The extracellular acidification rate (ECAR) and oxygen consumption rate (OCR) were determined using standard methods. Briefly, cells were seeded in XF96-well plates at a density of 2 × 10^4^/well. For glycolytic stress testing (Seahorse Bioscience, catalog no. 103020–100), 10 mmol/L glucose, 1 mmol/L oligomycin, and 50 mmol/L 2-deoxyglucose (2-DG) were used. For mitochondrial stress tests (Seahorse Bioscience, catalog no. 103015–100), 1 mmol/L oligomycin, 1 mmol/L FCCP, 0.5 mmol/L rotenone, and 0.5 mmol/L actinomycin A were used. Total cellular protein was used to normalize the measurements.

### Statistical Analysis

All statistical analyses were carried out using GraphPad Prism v.7.0. D’Agostino and Pearson omnibus normality test to determine whether the data are normally distributed. For two-group comparisons, two-tailed Student’s *t*-test was used for statistical analysis. After testing for normal distribution, statistical analysis was performed using ANOVA when more than two groups were compared. All data are presented as means ± SD (n.s., *p* > 0.05; *p ≤ 0.05; **p ≤ 0.01; ***,*p* ≤ 0.001).

## Results

### Increased GREM1 Expression in Intestinal Fibrosis

**FIGURE 1:**
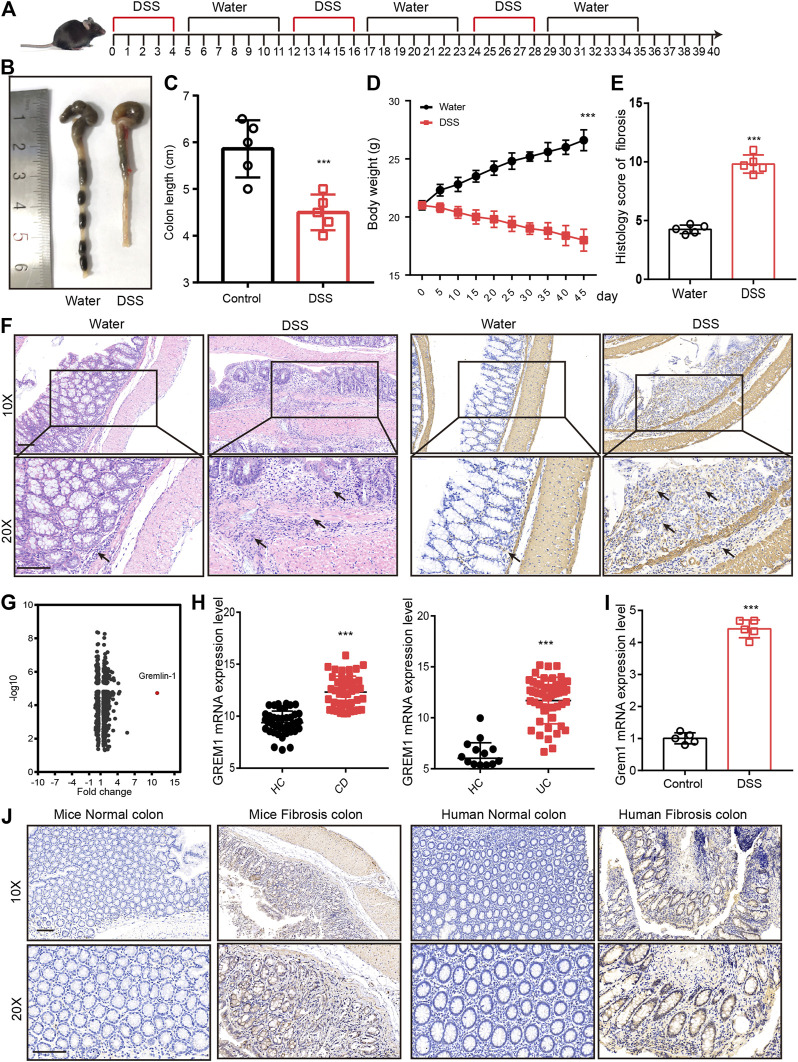
GREM1 expression increased in intestinal fibrosis. **(A)**. C57BL/6J mice were subjected to three cycles of 2.5% DSS for 5°days followed by 1 week of recuperation to establish mice intestinal fibrosis model. **(B)**. The appearance of the colons from the mice in control or DSS treated groups. **(C)**. The colon length statical results of mice from control or DSS treated group (*n* = 5). **(D)**. The body weight curve of mice received control or DSS treatment (*n* = 5), *p* values are derived from one-way ANOVA analysis. **(E)**. The histology score of intestinal fibrosis mice with different treatment. Fibrosis was quantified using a combined score of fibrosis severity, circularity and the extent of fibrosis (*n* = 5). **(F)**. The hematoxylin and eosin staining **(left panel)** and *α*-SMA IHC staining **(right panel)** of distal colon sections from control and DSS treated mice. scale bar 200 μm. **(G)**. The different expressed protein in DSS treatment mice and control mice that derived from proteomics analysis. **(H)**. The mRNA expression of GREM1 (human) in healthy control (HC) and IBD patient’s data from SRP077046 and SRP100787. **(I)**. The mRNA expression of *Grem1* in control and intestinal fibrosis mice (*n* = 5). **(J)**. IHC staining of GREM1 in both mice and human in intestinal fibrosis tissues. scale bar 200 μm. *p* values are derived from two-sided Student’s *t* test. **p ≤ 0.01; ***p ≤ 0.001.

To screen for potential therapeutic targets for intestinal fibrosis, we established an intestinal fibrosis mouse model by oral administration of three 2.5% DSS cycles to C57BL/6J mice (Figure 1A). The results showed that all DSS-treated mice developed serious colitis, and colon length and body weight were significantly lower in DSS-treated mice (Figures 1B–E). Further, intestinal tissue sections were subjected to hematoxylin-eosin (H&E) and anti-*α*-SMA immunohistochemical (IHC) staining. Pathological examination revealed that mice that received DSS treatment developed intestinal fibrosis, with fibroblast proliferation and ECM deposition (Figure 1F). Proteomic analysis of intestinal tissues indicated that GREM1 protein levels were dramatically elevated in fibrosed colon compared to that in normal colon tissue (Figure 1G). To confirm this in humans, we first compared *GREM1* mRNA expression using SRA datasets (SRP077046 and SRP100787) containing data for tissues from both healthy controls and CD and UC patients. The results showed that *GREM1* mRNA levels were significantly increased in human intestinal colitis samples (Figure 1H). Furthermore, we detected *GREM1* mRNA expression in intestinal fibrosis tissue and found that *GREM1* mRNA expression was also upregulated in fibrosed colon (Figure 1I). In line with this, IHC staining results revealed that GREM1 protein levels were greatly elevated in both murine and human intestinal fibrosis sections (Figure 1J). Collectively, these results suggested that GREM1 abundance was significantly increased in fibrosed colon tissues.

### GREM1 Promotes Intestinal Fibroblast Proliferation and Activation

**FIGURE 2:**
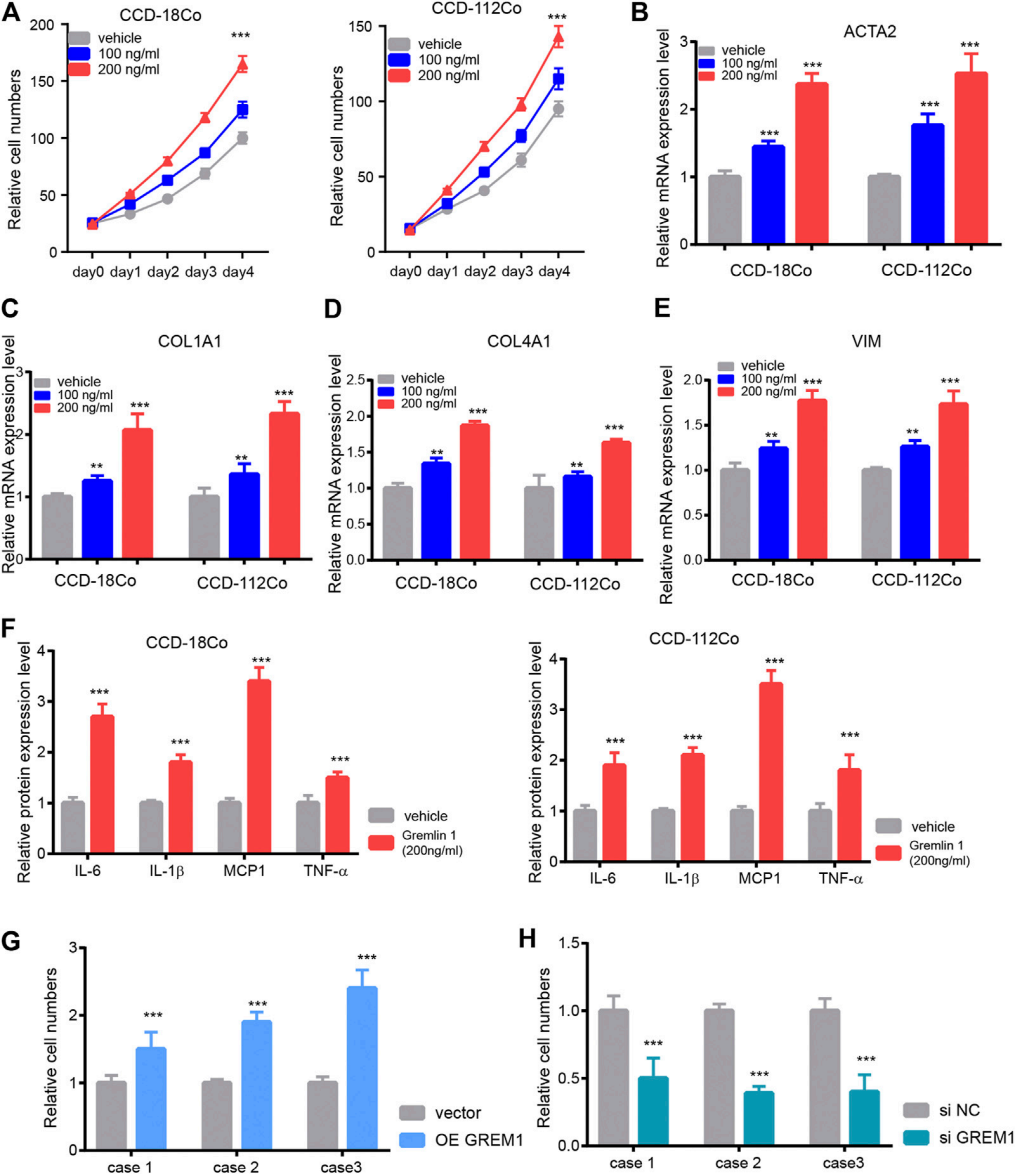
Gremlin 1 promotes the proliferation and activation of intestinal fibroblast. **(A)**. Cell viability assay of human intestinal fibroblast cells CCD-18Co and CCD-112Co upon different dose of Gremlin 1 (100 and 200 ng/ml) treatment. **(B–E)**. The Q-PCR results of ACTA2 **(B)**, COL1A1 **(C)**, COL4A1 **(D)** and VIM **(E)**, intestinal fibroblast cells CCD-18Co and CCD-112Co upon 100 and 200 ng/ml of Gremlin 1 treatment for 24 h. **(F)**. The foldchanges of inflammation cytokines in human fibroblast cells CCD-18Co and CCD-112Co upon 200 ng/ml Gremlin 1 treatment for 24 h. **(G)**. The system of fibroblast and intestinal organoid culture system. **(H–I)**. The relative IL-1*β* level in the culture medium of the human intestinal organoid upon co-cultured with GREM1 gene overexpressed fibroblast cell **(H)** or GREM1 gene knockdown fibroblast cell **(I)** for 48 h. These experiments were repeated twice. *p* values are derived from one-way ANOVA analysis. Dunnett’s test was used to analysis the difference between control group to the other groups. **p ≤ 0.01; ***p ≤ 0.001.

Next, we aimed to investigate the functional roles of GREM1 in the process of intestinal fibrosis development. Given that fibroblasts are the dominant effector cells in the development of intestinal fibrosis, we postulated whether GREM1 could promote proliferation and activation of intestinal fibroblasts. Two human intestinal fibroblast cell lines, CCD-18Co and CCD-112Co, were treated with different doses of the GREM1 protein in a serum-free culture medium. The results showed that GREM1 promoted fibroblast cell growth in a dose-dependent manner (Figure 2A). Further, to reveal whether GREM1 could facilitate the activation of fibroblasts, RT-qPCR was performed to detect altered expression of several typical fibroblast activation related genes, including *ACTA2*, *COL1A1, COL4A1*, and *VIM* in fibroblast cells upon administration of GREM1. Consistently, GREM1 treatment significantly increased the expression of these genes (Figures 2B–E). Given that activated fibroblasts could facilitate the progression of intestinal fibrosis by secreting cytokines, ELISA was performed to detect protein levels of IL-6, IL-1*β*, MCP1, and TNF. Levels of these cytokines were greatly increased after GREM1 treatment (Figure 2F). To further confirm this, intestinal fibroblast cell lines derived from patients with colon fibrosis were established following GREM1 ectopic expression or knockdown (Supplementary Figure S1). The results showed that additional GREM1 treatment significantly promoted the proliferation and activation of fibroblasts (Figure 2G). Conversely, the silencing of *GREM1* in fibroblasts delayed the fibrotic process (Figure 2H). Taken together, these data indicated that GREM1 could promote intestinal fibroblast proliferation and activation.

### GREM1 Promotes Intestinal Fibroblast Proliferation by Enhancing Fatty Acid Oxidation

**FIGURE 3:**
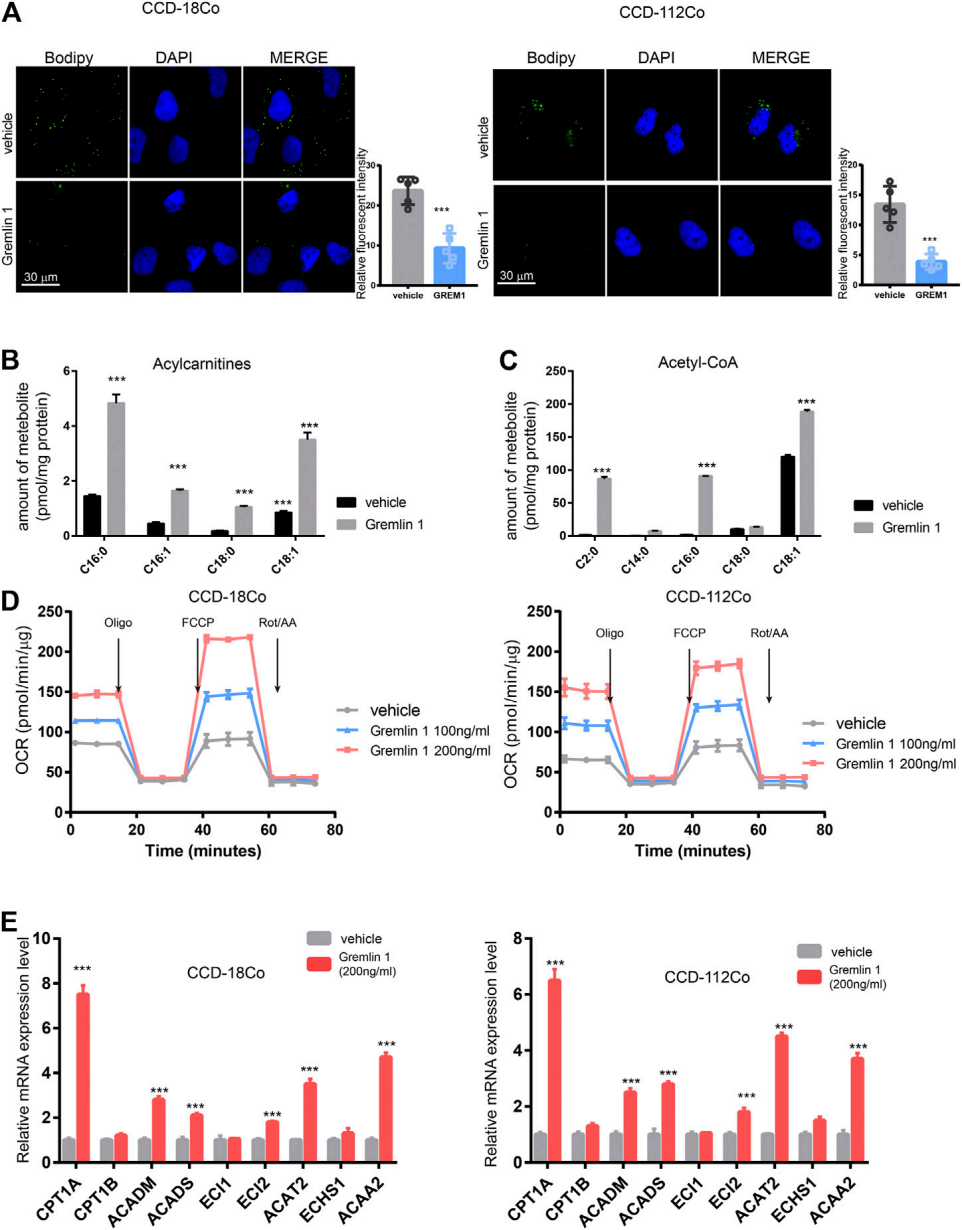
Gremlin 1 facility the FAO of intestinal fibroblast cell. **(A)**. Bodipy assay of human intestinal fibroblast cells CCD-18Co and CCD-112Co cells that treated with 200 ng/ml Gremlin 1 for 24 h or not. Relative fluorescent intensity was calculated of five random fields of slides **(B, C)**. The amount of representative individual lipid species, lipid droplets degraded production, Acylcarnitines **(B)** and Acetyl-CoA **(C)** in human intestinal fibroblast cells CCD-18Co and CCD-112Co cells treated with 200 ng/ml Gremlin 1 for 24 h or not. The amounts of acylcarnitine and acetyl-CoA (were normalized to the total protein of fibroblast cells. **(D)**. The measurement of OCR in human intestinal fibroblast cells CCD-18Co and CCD-112Co cells treated with 200 ng/ml Gremlin 1 for 24 h or not. **(E)**. Q-PCR results of the FAO related genes expression in human intestinal fibroblast cells CCD-18Co and CCD-112Co cells treated with 200 ng/ml Gremlin 1 for 24 h or not. These experiments were repeated twice *p* values are derived from two-sided Student’s *t* test. **p ≤ 0.01; ***p ≤ 0.001.

After stimulation with inflammatory effectors, fibroblasts start to proliferate and undergo trans-differentiation into contractile myofibroblasts, accompanied by energy status switching from quiescent to activated. In the kidney fibroblasts, Dario reported that Interleukin-1β activates a MYC-dependent metabolic switch in kidney stromal cells necessary for progressive tubulointerstitial fibrosis (Lemos et al., 2018). In addition, proceeding study reported that lipid metabolism in activated hepatic stellate cells is enhanced during liver fibrosis (Khomich et al., 2019); thus, we measured the lipid droplet content in GREM1-treated fibroblasts. BODIPY staining results showed that the abundance of lipid droplets was significantly decreased in GREM1 treated fibroblasts (Figure 3A). Next, the two major fatty acid oxidation products were measured in fibroblast cells treated with GREM1 or not. The results showed that acylcarnitine, derived from fatty acid oxidation by carnitine palmitoyl-transferase 1, (Schreurs et al., 2010; Qu et al., 2016), was significantly increased (Figure 3B). In line with this, the amounts of acetyl-CoA and fatty acid *β*-oxidation breakdown products were greatly elevated (Figure 3C). Furthermore, the OCR of GREM1-treated fibroblast cells was detected using a Seahorse. The data showed that GREM1 treatment markedly enhanced OCRs in fibroblast cells (Figure 3D, Supplementary Figure S2A). Moreover, GREM1 treatment did not affect the ECAR (Supplementary Figure S2B). Given that fatty acid oxidation produced much amount of energy, which could support the activation and proliferation of fibroblast. Furthermore, we aim to identify whether the promoting effects of GREM1 depended on enhanced FAO in fibroblasts. We firstly detected the expression of FAO related genes in fibroblast cells upon GREM1 treatment. The results showed that *CPT1A, ACADM, ACADS ECI2 ACAT2,* and *ACAA2* expression significantly increased upon GREM1 treatment (Figure 3E). Further, we blocked the FAO in mitochondria using Etomoxir, an inhibitor of FAO. As expected, the indicated that Etomoxir usage impeded the proliferation and activation of fibroblasts otherwise mediated by GREM1 treatment (Supplementary Figure S3). Collectively, GREM1 promoted intestinal fibroblast proliferation by enhancing fatty acid oxidation.

### GREM1 Activated VEGFR-2 Receptor in Fibroblasts

**FIGURE 4:**
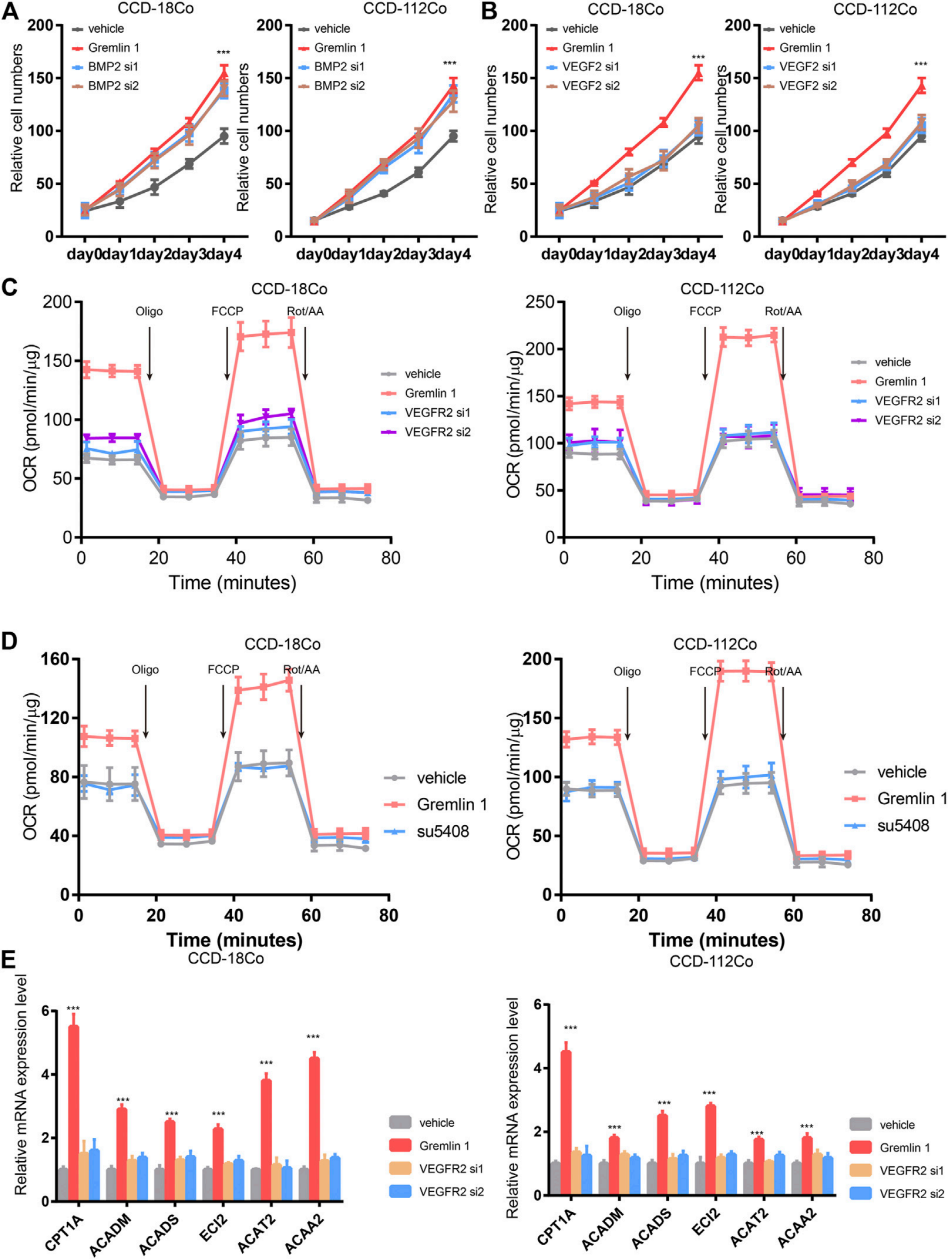
Gremlin 1 activated VEGFR-2 to enhance the FAO of fibroblast. **(A)**. Cell viability assay of human intestinal fibroblast cells CCD-18Co and CCD-112Co treated with 200 ng/ml Gremlin 1 with or with not silencing BMP2 with siRNA. **(B)**. Cell viability assay of human intestinal fibroblast cells CCD-18Co and CCD-112Co treated with 200 ng/ml Gremlin 1 with or with not silencing VEGFR-2 with siRNA. **(C)**. The OCR results of CCD-18Co and CCD-112Co cells treated with 200 ng/ml Gremlin 1 for 24 h, following silencing VEGFR-2 with siRNA or not. **(D)**. The OCR results of human intestinal fibroblast cells CCD-18Co and CCD-112Co cells treated with 200 ng/ml Gremlin 1, following VEGFR-2 inhibitor SU5408 (100 nM) treatment for 24 h. **(E)**. Q-PCR results of genes related with FAO metabolism in the human intestinal fibroblast cells CCD-18Co and CCD-112Co cells. *p* values are derived from two-sided Student’s *t* test. **p ≤ 0.01; ***p ≤ 0.001.

Next, we aimed to identify the receptor that senses GREM1 stimulation. Given that GREM1 is reported to be a BMP2 antagonist and ligand of VEGFR-2, we detected fibroblast cell proliferation upon knockdown of BMP2 or VEGFR-2 using siRNA (Figures 4A,B). The data showed that silencing VEGFR-2 expression dramatically diminished the growth-promoting effects of GREM1 in fibroblast cells, which was not observed in BMP2-silenced fibroblast cells, indicating that VEGFR-2 may act as a GREM1 receptor in intestinal fibroblasts. To further confirm this, VEGFR-2 was inhibited using the specific inhibitor SU5408. As anticipated, SU5408 also blocked the growth-promoting effects of GREM1 in fibroblasts (Supplementary Figure S4). Next, we measured OCRs to determine whether VEGFR-2 inhibition could block the enhanced FAO in fibroblasts. The data showed that both genetic and pharmacological VEGFR-2 inhibition could decrease the enhanced FAO mediated by GREM1 treatment (Figures 4C,D). Additionally, FAO-related mRNAs were also measured, and the results showed that expression of such genes decreased with VEGFR-2 inhibition (Figure 4E).

Taken together, GREM1 promoted intestinal fibroblast proliferation by activating VEGFR-2 receptor.

### GREM1 Activates MAPK Pathway to Enhance Fatty Acid Oxidation

**FIGURE 5:**
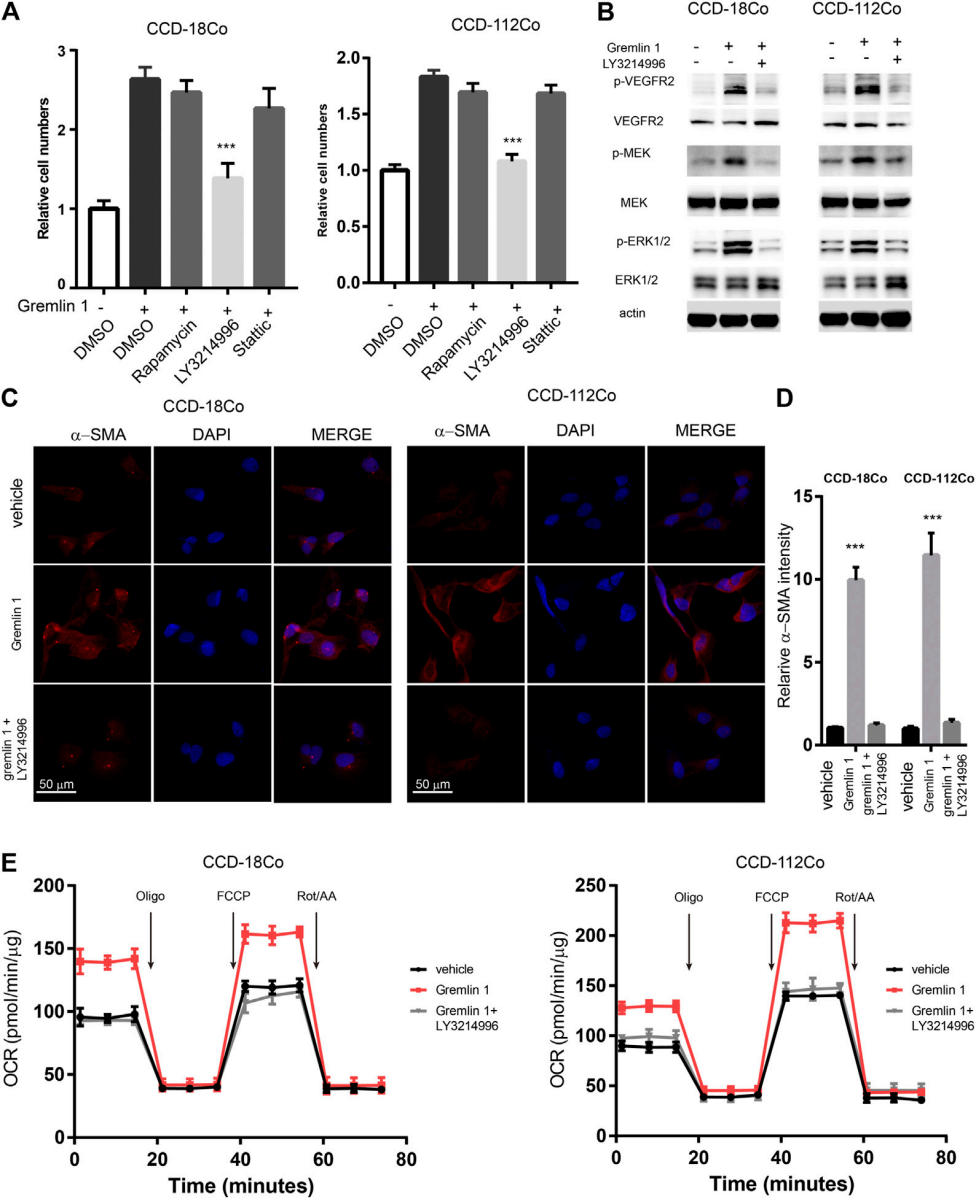
VEGFR2-MEK-ERK axis mediated the enhancement of FAO from Gremlin 1. **(A)**. Cell viability assay of human intestinal fibroblast cells CCD-18Co and CCD-112Co cells treated with 200 ng/ml Gremlin 1 and Rapamycin (10 nM), LY3214996 (10 nM) or stattic (10 μM) for 24 h. **(B)**. Immunoblotting analysis of VEGFR2-MEK-ERK signaling in human intestinal fibroblast cells CCD-18Co and CCD-112Co cells treated with 200 ng/ml Gremlin 1 and LY3214996 (10 nM) for 24 h. Actin acted as loading control and re-used for illustrative purposes. **(C)**. Immunofluorescence staining of a-SMA in human intestinal fibroblast cells CCD-18Co and CCD-112Co cells treated with 200 ng/ml Gremlin 1 and LY3214996 (10 nM). **(D)**. The intensity statistical results of a-SMA immunofluorescence staining from five random fields of slides. E. OCR measurement of CCD-18Co and CCD-112Co cells treated with 200 ng/ml Gremlin 1 and LY3214996 (10 nM) for 24 h. *p* values are derived from one-way ANOVA analysis. Dunnett’s test was used to analysis the difference between control group to the other groups. **p ≤ 0.01; ***p ≤ 0.001.

In order to uncover the underlying signaling mechanisms leading to FAO enhancement, the reported VEGFR-2 downstream signaling pathways (including the PI3K-mTOR, MAPK, and STAT3 pathways) were inhibited with the specific inhibitors Rapamycin, LY3214996, and Stattic, respectively, (Figure 5A). Cell viability assays showed that only the MAPK signaling inhibitor LY3214996 could diminish the otherwise increased proliferation mediated by GREM1. Western blot analysis showed that treatment with GREM1 strongly increased the phosphorylation levels of p-VEGFR-2, its downstream signaling p-MEK, and p-ERK1/2, which were inhibited by LY3214996 treatment (Figure 5B). Immunofluorescent imaging also revealed that MAPK signaling inhibition blocked increased *α*-SMA expression in intestinal fibroblasts (Figures 5C,D). Furthermore, we measured OCR levels in intestinal fibroblasts. Accordingly, the elevated OCRs were abolished by LY3214996 treatment (Figure 5E). In line with this, the increased acylcarnitine and acetyl-CoA levels decreased upon LY3214996 treatment (Supplementary Figure S5). Together, these data sets suggested that GREM1 activated the MAPK pathway to enhance fatty acid oxidation in intestinal fibroblasts.

### Blocking GREM1-VEGFR2 Axis Impedes Intestinal Fibrosis Progression *in vivo*

**FIGURE 6:**
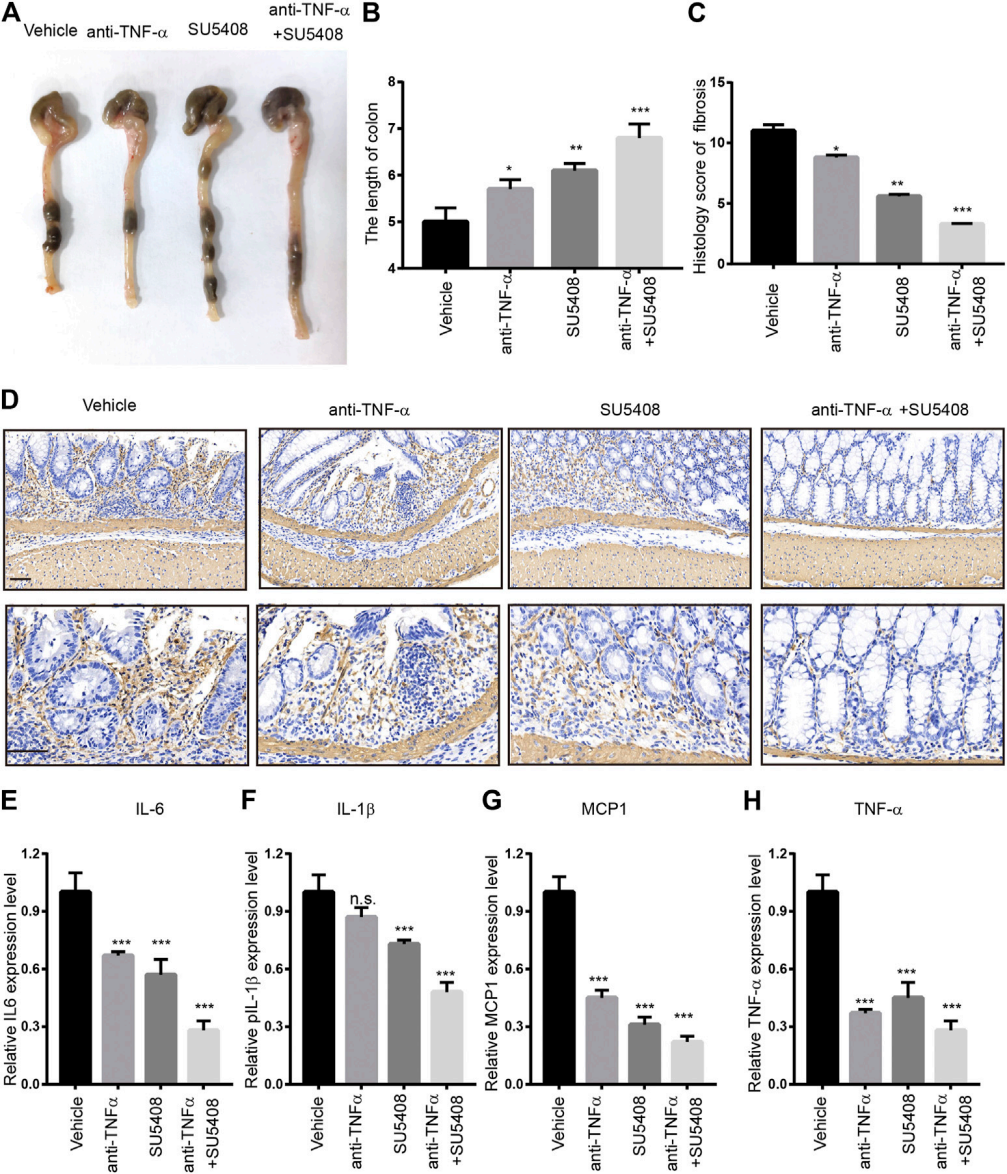
Gremlin 1-VEGFR2 axis disruption impeded the progression of intestinal fibrosis. **(A)**. The appearance of the colons from the mice with intestinal fibrosis treated with vehicle, anti-TNF-*α* monotherapy (100 μg), SU5408 (5 mg/kg), or anti-TNF-α therapy combined with SU5408 treatment. **(B)**. The statistical results of colon length in mice with vehicle, anti-TNF-α monotherapy (100 μg), SU5408 (5 mg/kg), or anti-TNF-α therapy combined with SU5408 treatment (*n* = 5). **(C)**. The histology score of intestinal fibrosis mice with different treatment. Fibrosis was quantified using a combined score of fibrosis severity, circularity and the extent of fibrosis (*n* = 5). **(D)**. *α*-SMA IHC staining of intestinal sections from intestinal fibrosis mice with different treatment. scale bar 100 μm **(E–H)**. The protein expression level of IL-6, IL-1*β*, MCP1, and TNF-*α* in the colon of different group mice treated with vehicle, anti-TNF-α monotherapy (100 μg), SU5408 (5 mg/kg), or anti-TNF-*α* therapy combined with SU5408 treatment (*n* = 5). *p* values are derived from one-way ANOVA analysis. Dunnett’s test was used to analysis the difference between control group to the other groups. n. s., *p* > 0.05; *p ≤ 0.05; **p ≤ 0.01; ***p ≤ 0.001.

Next, we evaluated whether GREM1-VEGFR2 axis downstream signaling blockage by a VEGFR2-specific inhibitor could prevent intestinal fibrosis progression upon DSS challenge. Anti-TNFα therapy is one of standard treatment strategies for intestinal fibrosis. We aim to determine whether combine SU5408 with Anti-TNF therapy could provide more benefits for intestinal fibrosis patients. For this purpose, either VEGFR2-specific inhibitor SU5408 or SU5408 plus anti-TNF-α therapy was applied to the intestinal fibrosis mouse model (Figure 6A). The results showed that SU5408 usage could restore the duration of colon reduction mediated by DSS challenge, while anti-TNF-α monotherapy was associated with a slight improvement in colonic length. Of note, combining anti-TNF-α antibody with SU5408 significantly restored colonic length (Figures 6B,C). Furthermore, the deposition of ECM was found to be dramatically reduced in both groups of mice treated with SU5408 alone or in combination with anti-TNF-α therapy. In addition, *α*-SMA IHC staining intensity was much weaker in colon sections derived from SU5048-treated mice than in mice in the control group, suggesting a reduction in the proportion of fibroblasts (Figure 6D). Additionally, we measured *Col1a1* and *Vim* mRNA expression levels in colon. GREM1-VEGFR2 axis blockage treatment greatly inhibited the expression of *Col1a1* and *Vim* (Supplementary Figure 6). Moreover, VEGFR2 inhibition significantly attenuated the levels of the inflammation-related cytokines TNF-α, MCP1, IL-6, and IL-1β (Figures 6E–H). Together, these data sets indicated that GREM1-VEGFR2 axis disruption could diminish the proliferation and function of fibroblasts in the colon of mice with intestinal fibrosis.

## Discussion

In this study, we found that GREM1 levels were greatly increased in both human and murine fibrosed colon. Functional experiments revealed that GREM1 promoted intestinal fibroblast cell proliferation by enhancing fatty acid oxidation. Further mechanistic studies revealed that GREM1 could activate VEGFR2 and trigger downstream MAPK signaling, which facilitated the expression of FAO-related genes, consequently enhancing fatty acid oxidation.

Intestinal fibrosis is a common finding in patients with IBD (Latella et al., 2014). Current studies indicate that only anti-inflammatory therapy does not impede the development of fibrosis (Latella and Papi, 2012). Fibroblast expansion and activation are major factors that facilitate the progression of intestinal fibrosis. Rapid growth and ECM deposition require a huge amount of energy. Previous studies reported that fibroblasts could reprogram their metabolism by enhancing glycolysis and FAO to sustain cell proliferation and activation (Schreurs et al., 2010; Latella and Papi, 2012; Latella et al., 2014; Qu et al., 2016; Khomich et al., 2019; Chen et al., 2020). In kidney stromal cells, Interleukin-1*β* induced glycolysis enhancement is necessary for progressive tubulointerstitial fibrosis (Lemos et al., 2018). Additionally, hepatic stellate cell activation results in decrease of lipid droplet abundance and increased levels of polyunsaturated fatty acids (Tuohetahuntila et al., 2015). We consistently observed significant numbers of lipid droplets and enhanced FAO in intestinal fibroblast cells upon GREM1 treatment. On the other hand, Chung et al. (Chen et al., 2020) reported that the expression of FAO-associated enzymes in renal tubule epithelium is reduced in aged rat kidneys. This facilitates the progression of renal fibrosis indicating that FAO alterations in fibroblast and epithelial cells affect the progression of fibrosis differently.

Recent studies have shown that GREM1 is mainly secreted by fibroblast cells, including cancer-associated fibroblasts (Worthley et al., 2015) which can promote the proliferation of cancer cells (Sneddon et al., 2006). High GREM1 expression is related to poorer outcomes in CRC and breast cancer patients (Dutton et al., 2019; Neckmann et al., 2019; Ren et al., 2019). GREM1 also regulates differentiation of glioma cells (Yan et al., 2014). In the current study, we found that GREM1 could activate VEGFR 2, but is not involved in BMP signaling, which further activated downstream MAPK signaling, resulting in FAO enhancement. Recently, GREM1 was reported to activate STAT3 signaling in breast cancer cells (Sung et al., 2020). However, our data suggested that STAT3 was not involved in GREM1-mediated fibroblast cell proliferation and activation, indicating that the signaling regulated by GREM1 is largely context-dependent.

Our *in vivo* experimental results showed that disruption to the GREM1-VEGFR2 axis downstream signaling using SU5408 could delay the progression of intestinal fibrosis. Recently, Kobayashi and colleagues reported that a GREM1-neutralizing antibody could prevent colorectal carcinogenesis (Kobayashi et al., 2020). We realized that a GREM1-neutralizing antibody might be a superior option for our animal studies. However, such a neutralizing antibody is commercially inaccessible. In addition, after carefully checking the patent, such a GREM1-neutralizing antibody was designed to block interactions involving GREM1 and BMP proteins, indicating that this antibody is not the best choice for blocking the GREM1 and VEGFR2 axis. Thus, further efforts are needed to develop neutralizing antibodies or peptides that block interactions between GREM1 and VEGFR2. In addition, our current data showed that blocking the GREM1–VEGFR2 axis combined with anti-TNF-*α* therapy could provide benefits in mice suffering from intestinal fibrosis, indicating that the combine therapy of anti-TNF-*α* and GREM1–VEGFR2 blockage could be a potential strategy for intestinal fibrosis patients.

In conclusion, our data revealed that GREM1 promoted the proliferation and activation of fibroblasts by activating VEGFR-2, leading to FAO enhancement. Inhibition of the GREM1-VEGFR2 axis impeded progression in the murine intestinal fibrosis model, indicating that GREM1 could represent a potential target for anti-fibrotic therapy.

## Data Availability

The raw data supporting the conclusions of this article will be made available by the authors, without undue reservation, to any qualified researcher.
