# Supplementary material for: Targeting Gremlin 1 Prevents Intestinal Fibrosis Progression by Inhibiting the Fatty Acid Oxidation of Fibroblast Cells
Source: Front Pharmacol. 2021 Apr 22;12:663774. doi: 10.3389/fphar.2021.663774 (PMC8100665; doi:10.3389/fphar.2021.663774)
Supplement: Supplementary file 1 [file datasheet3.docx]

Supplementary figure 1


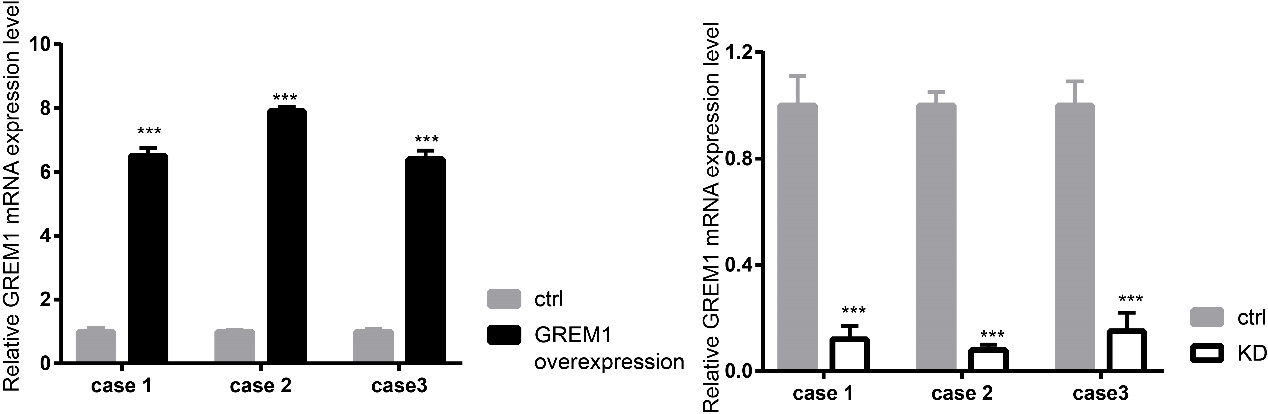


The Q-PCR analysis of GREM1 overexpression and knockdown results in the intestinal fibroblast cells derived from human fibrosis colon

Supplementary figure 2


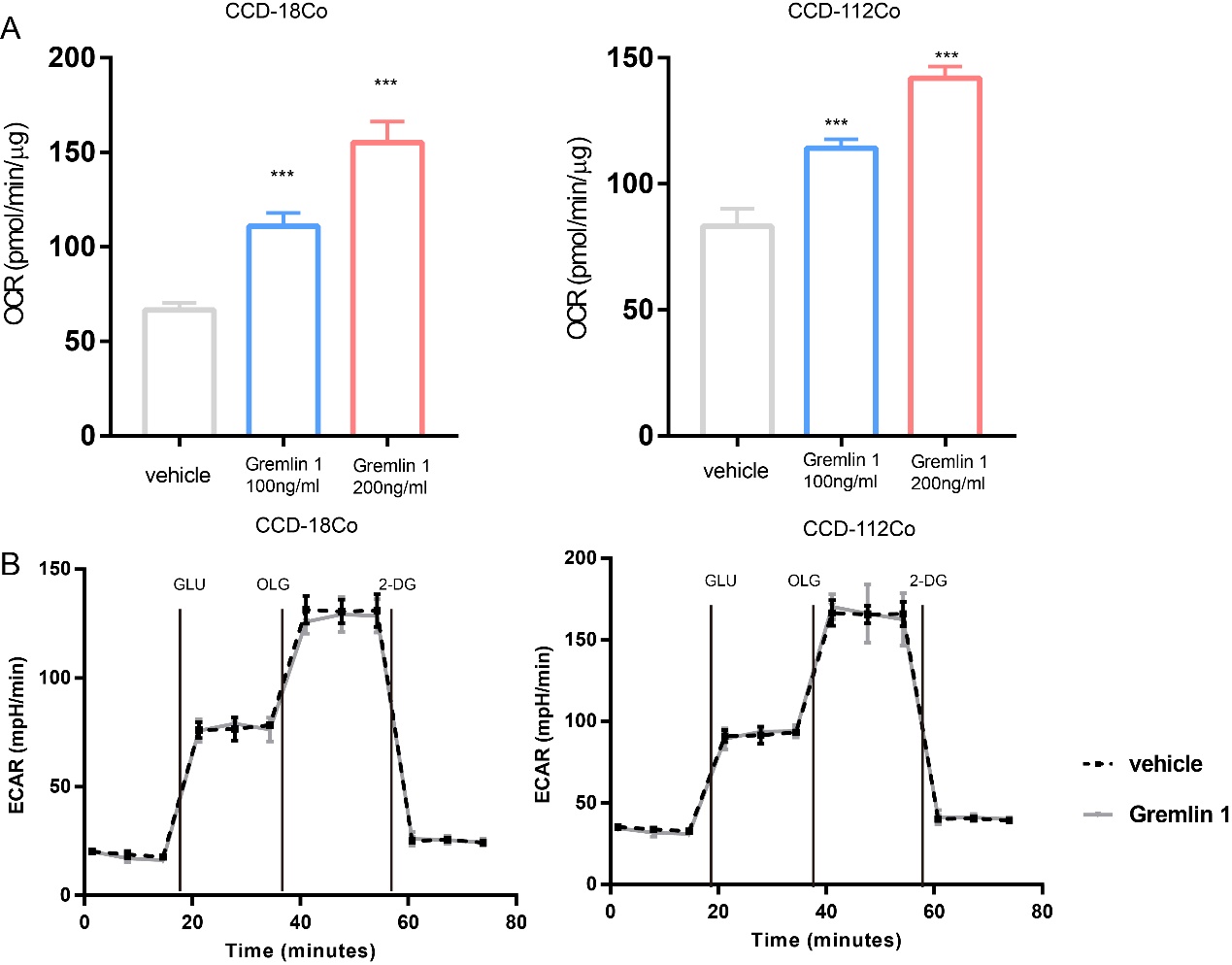


The basal OCR(A) and ECAR(B) in CCD-18Co and CCD-112Co cells treated with Gremlin 1(200 ng/ml) or not for 24h.

Supplementary figure 3


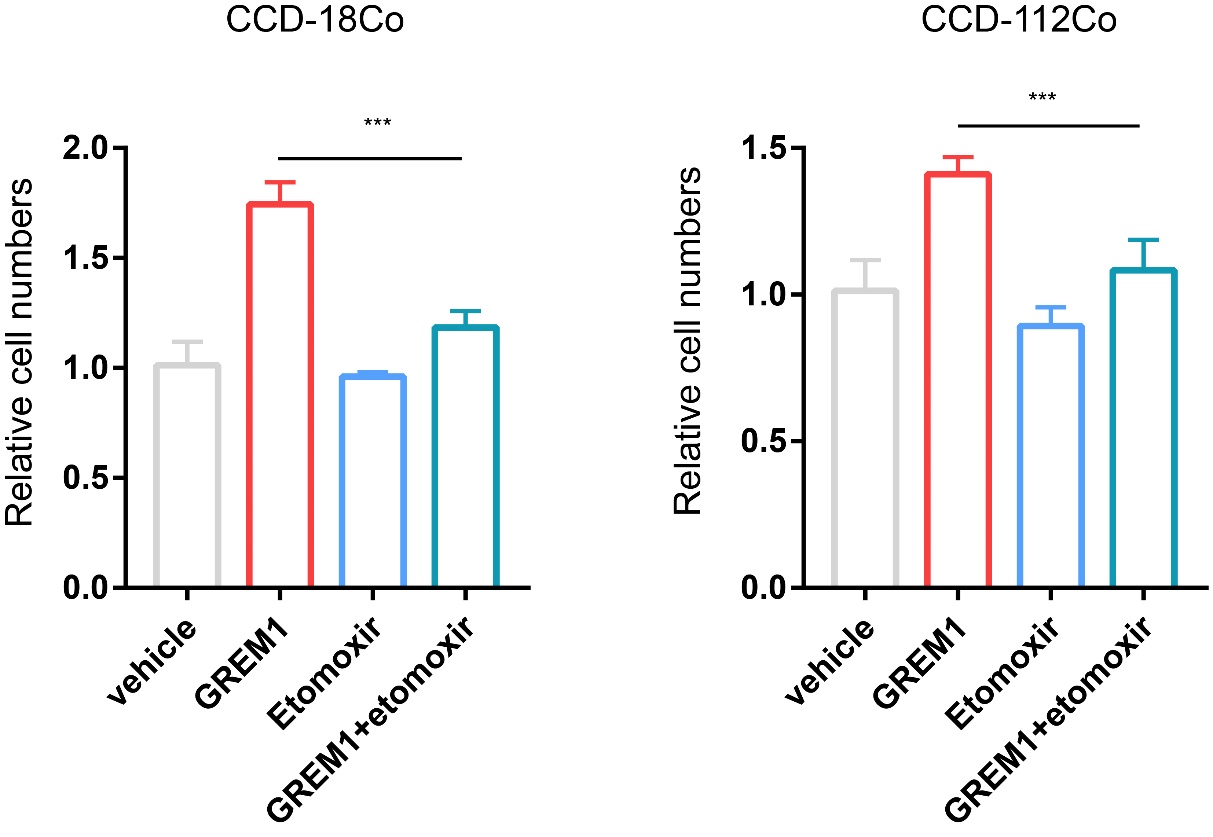


Cell viability assay of CCD-18Co and CCD-112Co treated with Gremlin 1(200 ng/ml) with or without Etomoxir (100 μm) treatment for 24h

Supplementary figure 4


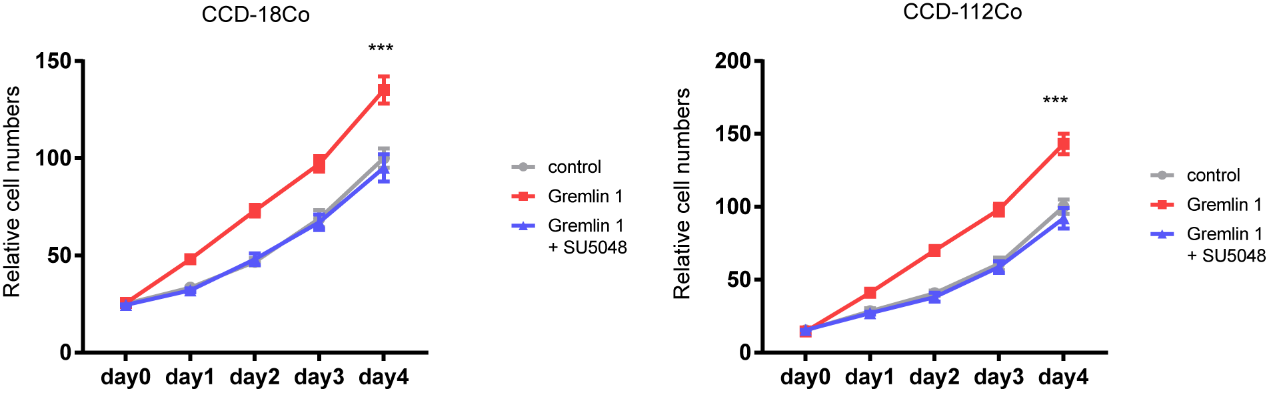


Cell viability assay of CCD-18Co and CCD-112Co treated with Gremlin 1(200 ng/ml) with or without SU5408 (100 nm) treatment for 24h.

Supplementary figure 5


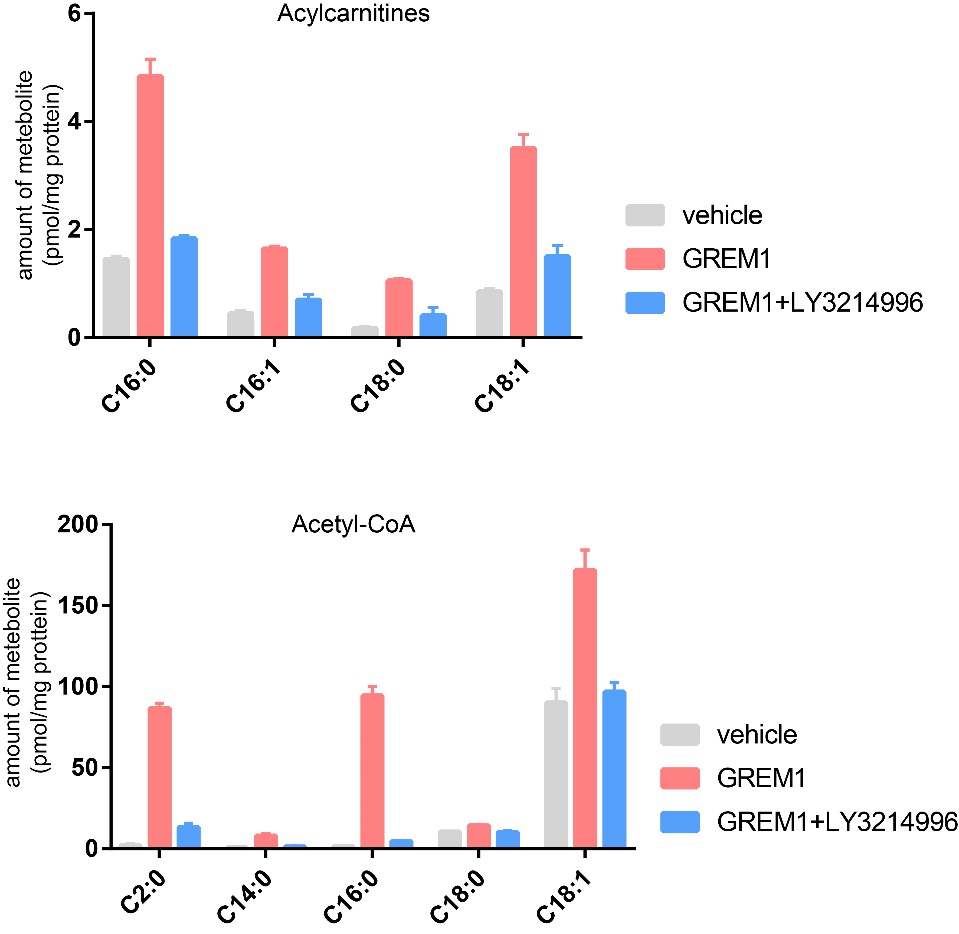


Acylcarnitines and Acetyl-CoA in human intestinal fibroblast cells CCD-18Co and CCD-112Co cells treated with 200 ng/mL Gremlin 1 for 24h combined with LY3214996 or not.

Supplementary figure 6


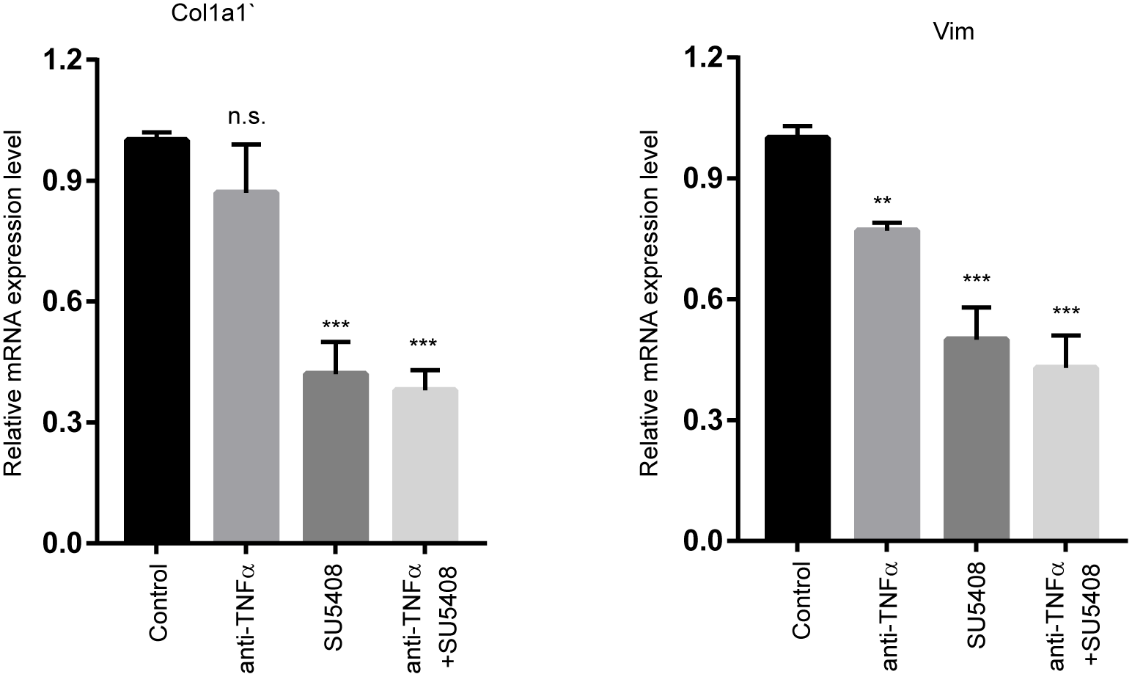


The Q-PCR results of COL1A1 and VIM in the colon of different group mice treated with vehicle, anti-TNF-α monotherapy (100 μg), SU5408 (5mg/kg), or anti-TNF-α therapy combined with SU5408 treatment.

**SiRNA sequence**

GREM1-si 1 CATCGATTTGGATTAAGCC

GREM1-si 2 TCGATGGATATGCAACGA

BMP2-si 1 CCCGGAGATTCTTCTTTAA

BMP2-si 2 CCACTTGGAGGAGAAACAA

Vegfr2 si1 GCGATGGCCTCTTCTGTAA

Vegfr2 si1 GGAATTGACAAGACAGCAA

Q-PCR primers

| Target name | Forward sequence | Reverse sequence |
| --- | --- | --- |
| GREM1（human） | CGGAGCGCAAATACCTGAAG | GGTTGATGATGGTGCGACTGT |
| Grem1(mice) | GGGACCCTACTGCCAACAG | TTTGCACCAATCTCGCTTCAG |
| Cpt1A | CCTGGGCATGATTGCAAAG | ACGCCACTCACGATGTTCTTC |
| Cpt1B | TCTTCCTGAACTGGCTGTCA | GTACCCACCATGCACTACCA |
| ACADM | ACAGGGGTTCAGACTGCTATT | TCCTCCGTTGGTTATCCACAT |
| ACADS | CGGCAGTTACACACCATCTAC | GCAATGGGAAACAACTCCTTCTC |
| ECI1 | CTGCGGTTGTACCAGTCCAA | GATGCGGTAGTCACAGGTCA |
| ECI2 | ATGGGACGCATGGAATGCC | TTCAAACCCAGTTGATTTCCTGT |
| ACAT2 | GCGGACCATCATAGGTTCCTT | ACTGGCTTGTCTAACAGGATTCT |
| ECHS1 | TGAGCTTGCCATGATGTGTGA | AACAGGACAAATCTTGCTGACA |
| ACAA2 | CTGCTCCGAGGTGTGTTTGTA | GGCAGCAAATTCAGACAAGTCA |
