# Supplementary figures and images for: Targeting Gremlin 1 Prevents Intestinal Fibrosis Progression by Inhibiting the Fatty Acid Oxidation of Fibroblast Cells
Source: Front Pharmacol. 2021 Apr 22;12:663774. doi: 10.3389/fphar.2021.663774 (PMC8100665; doi:10.3389/fphar.2021.663774)

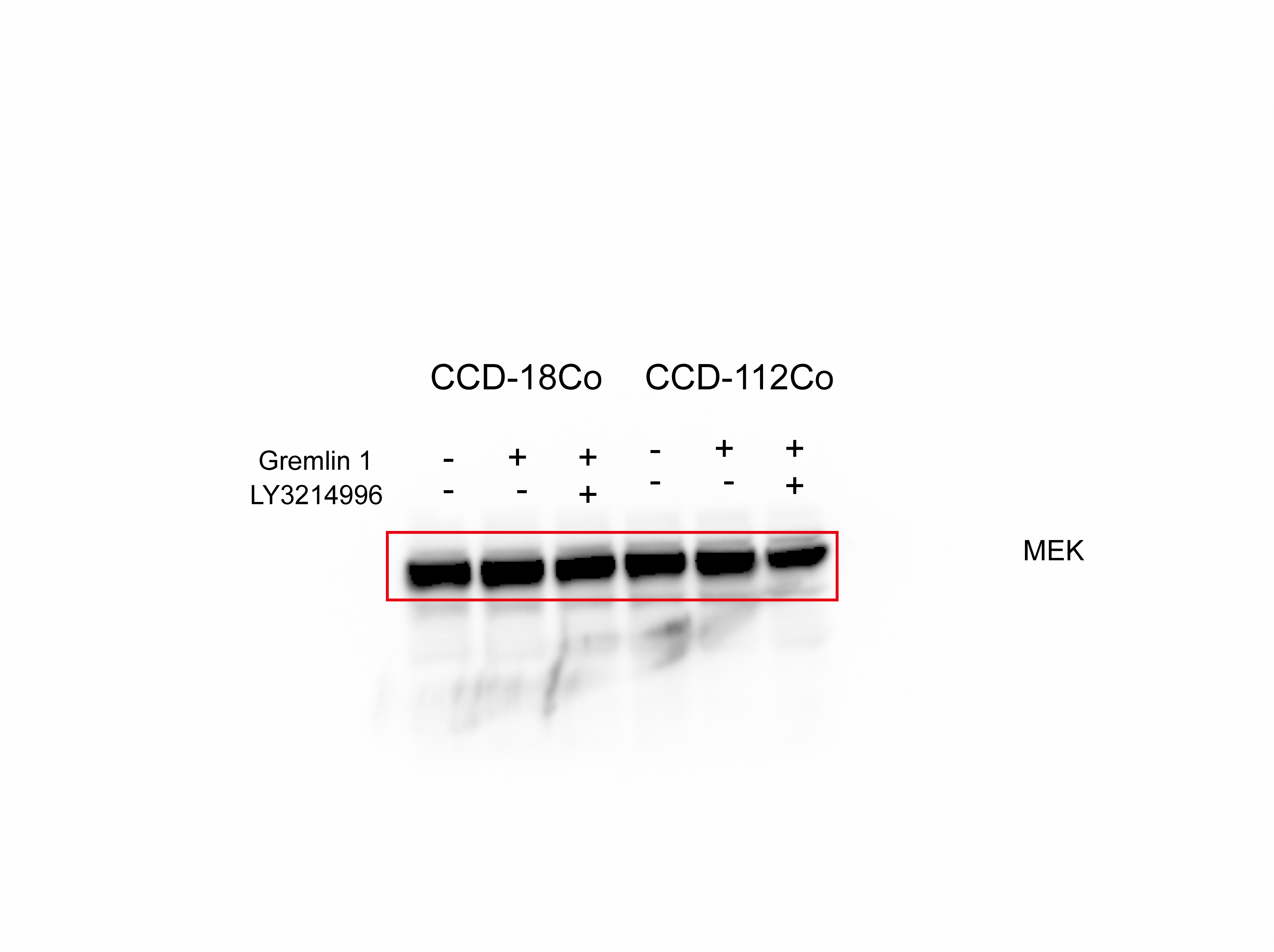

Supplement: Supplementary file 2 [file datasheet1.zip › wb raw data/MEK.tif]

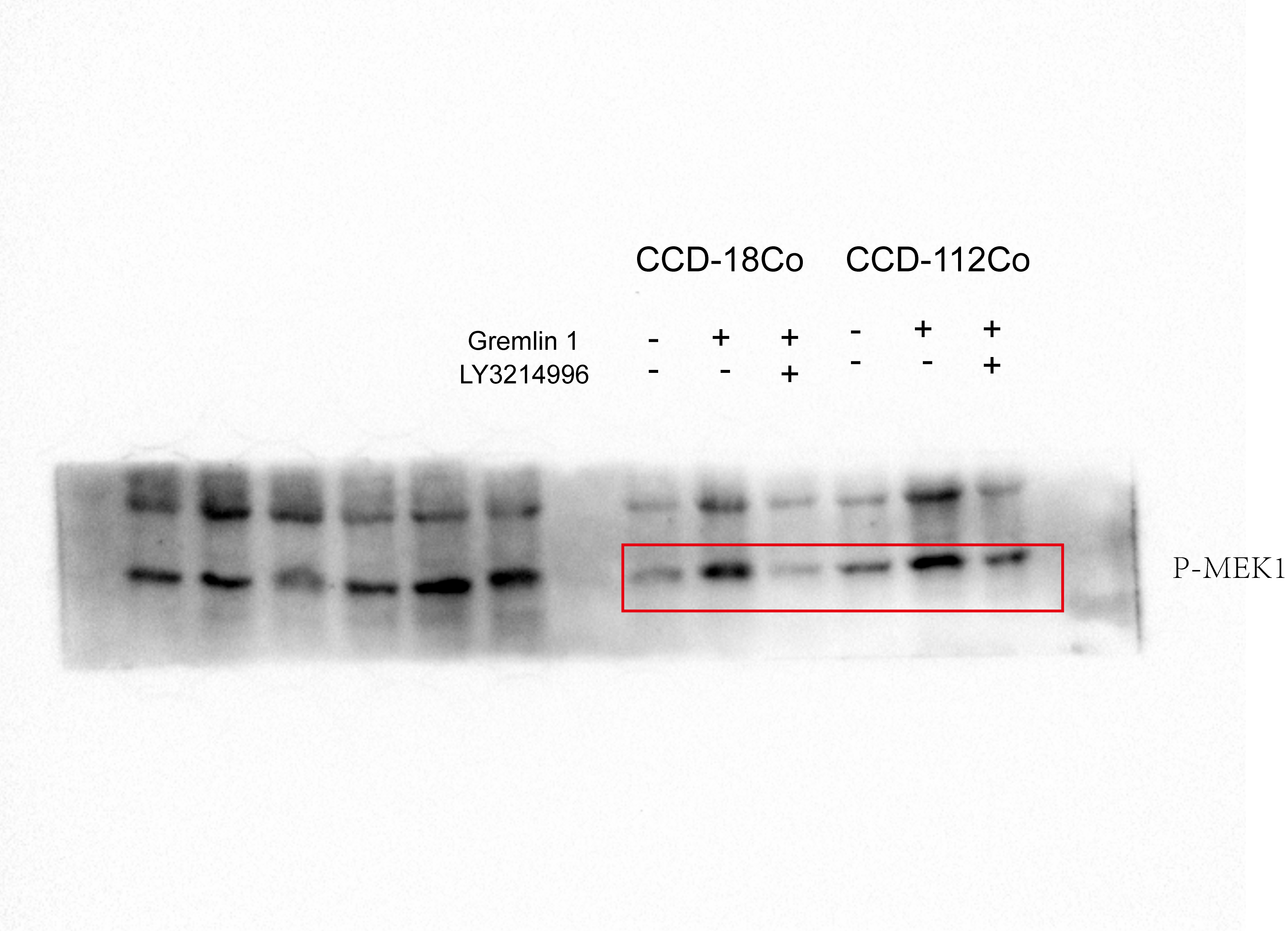

Supplement: Supplementary file 2 [file datasheet1.zip › wb raw data/P-MEK1.tif]

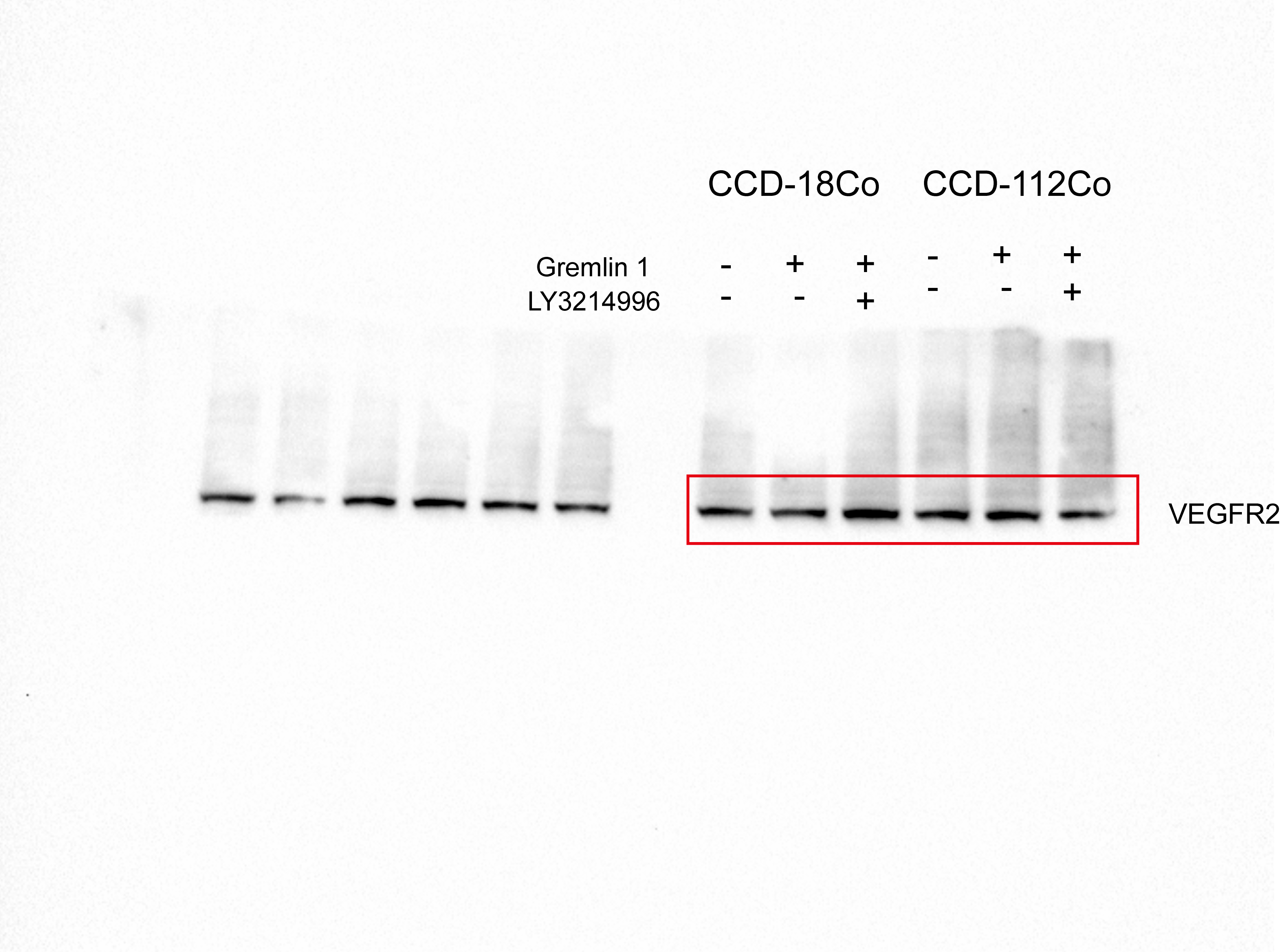

Supplement: Supplementary file 2 [file datasheet1.zip › wb raw data/VEGFR2.tif]

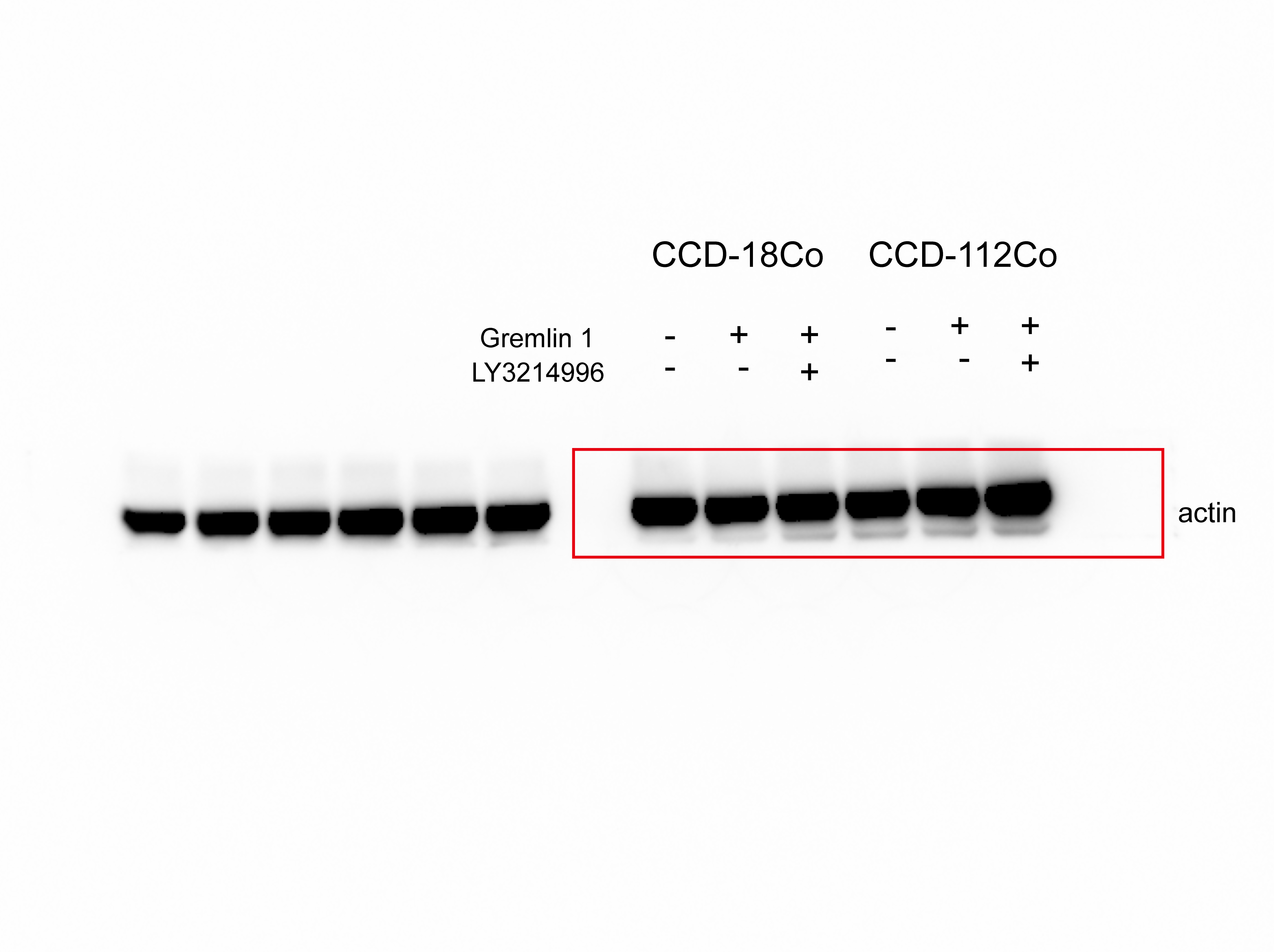

Supplement: Supplementary file 2 [file datasheet1.zip › wb raw data/actin.tif]

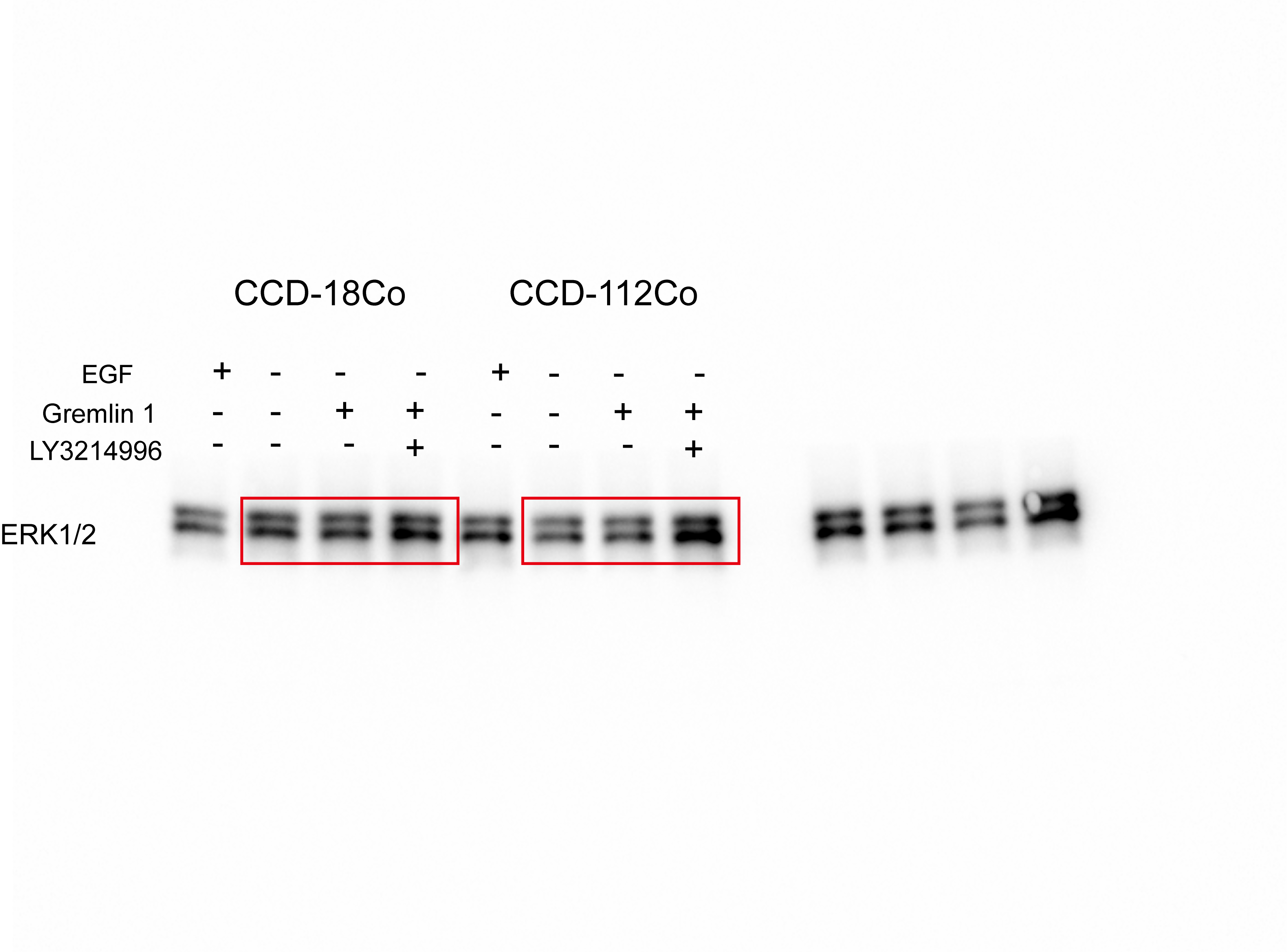

Supplement: Supplementary file 2 [file datasheet1.zip › wb raw data/erk1and2.tif]

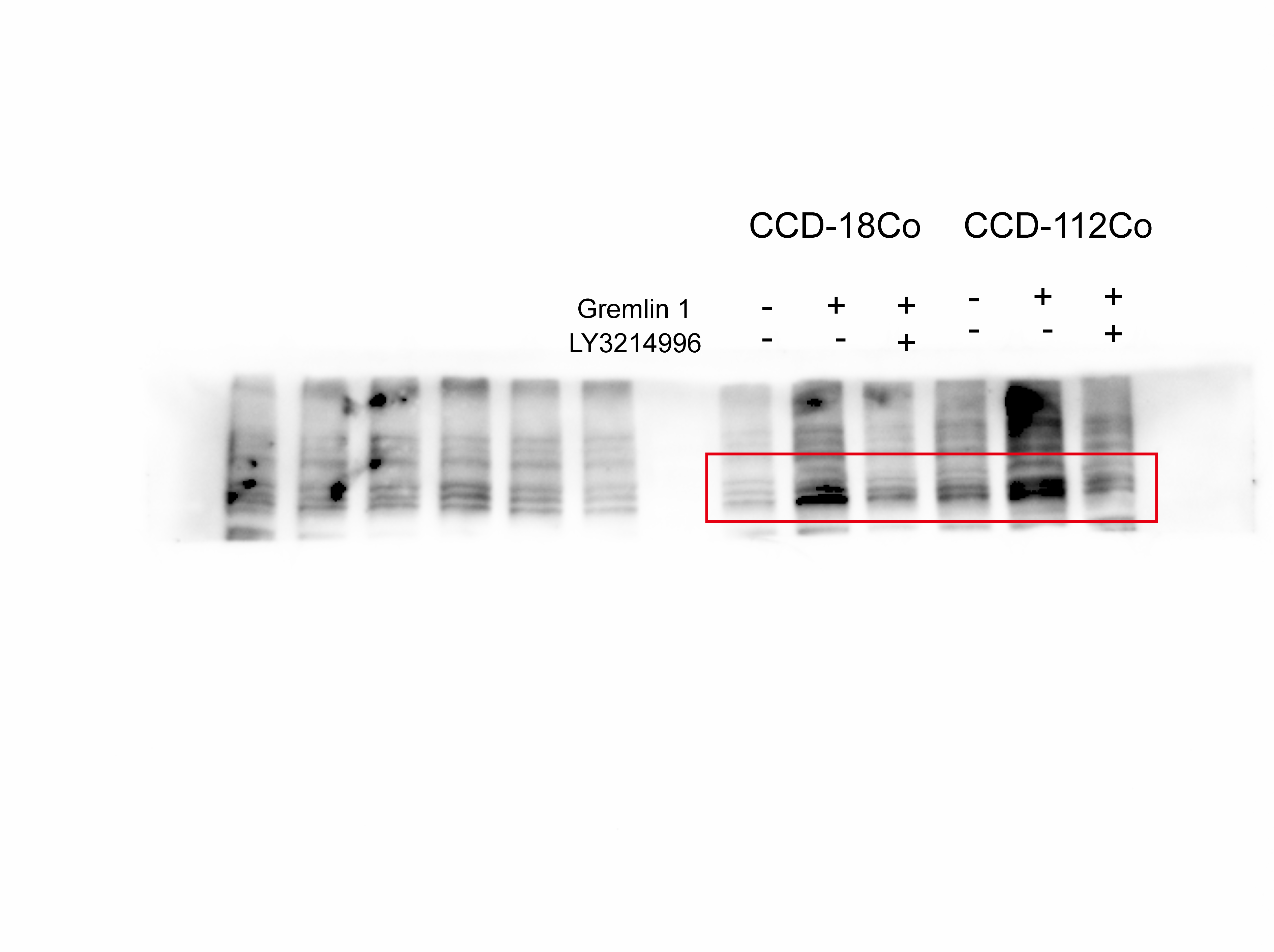

Supplement: Supplementary file 2 [file datasheet1.zip › wb raw data/p-VEGFR2.tif]

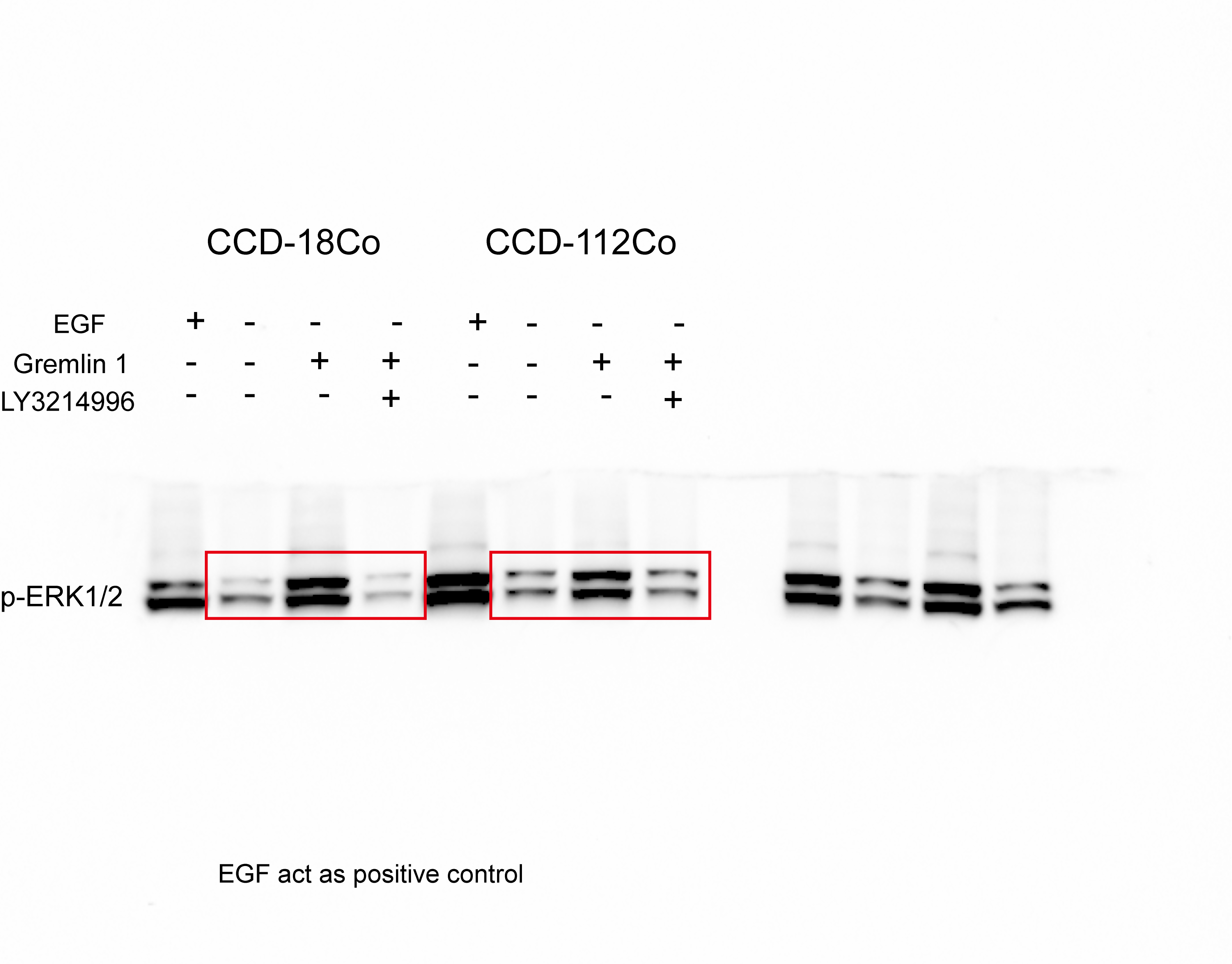

Supplement: Supplementary file 2 [file datasheet1.zip › wb raw data/p-erk1and2.tif]
